# Hsp90 is a potential risk factor for ovarian cancer prognosis: an evidence of a Chinese clinical center

**DOI:** 10.1186/s12885-023-10929-9

**Published:** 2023-05-31

**Authors:** Cancan Duan, KuoKuo Li, Xiaohua Pan, Zhaolian Wei, Lan Xiao

**Affiliations:** 1 Department of Obstetrics and Gynecology, The First Affiliated Hospital of Anhui Medical University, 218Th Jixi Road, Hefei, 230022 P.R. China; 2 NHC Key Laboratory of Study On Abnormal Gametes and Reproductive Tract (Anhui Medical University), Hefei, China

**Keywords:** OC, Hsp90, Prognosis, Immunohistochemistry, Enzyme-linked immunosorbent assay

## Abstract

**Background:**

The potential treatment effects of heat shock protein 90 (Hsp90) inhibitors in ovarian cancer (OC) are controversial. This research aims to investigate the relationship between the level of Hsp90 in peripheral blood and the prognosis of OC patients, as well as the clinicopathological indicators.

**Materials and methods:**

We retrospectively collected the clinicopathological indicators of OC patients who were admitted to the Department of Obstetrics and Gynecology of the First Affiliated Hospital of Anhui Medical University from 2017 to 2022. Hsp90 level in patient blood was detected by enzyme-linked immunosorbent assay, and the correlation between Hsp90 level and OC prognosis was systematically investigated. Kaplan–Meier method was used to draw the survival curve, and the average survival time and survival rate were calculated. The log-rank test and Cox model were used for univariate survival analysis, and the Cox proportional hazards model was applied for multivariate survival analysis. Based on the TCGA dataset of OC obtained by cBioPortal, Pearson’s correlation coefficients between Hsp90 level values and other mRNA expression values were calculated to further conduct bioinformatics analysis. GSEA and GSVA analysis were also conducted for gene functional enrichment. The expression of Hsp90 in OC tissues were evaluated and compared by Immunohistochemical staining.

**Results:**

According to the established screening criteria, 106 patients were selected. The enzyme-linked immunosorbent assay results showed that 50.94% OC patients with abnormal Hsp90 level. According to the outcome of Kaplan–Meier curves, the results revealed that the abnormal level of Hsp90 was suggested to poor prognosis (*P* = 0.001) of OC patients. Furthermore, the result of multivariate Cox proportional hazards regression model analysis also predicted that abnormal Hsp90 level (HR = 2.838, 95%CI = 1.139–7.069, *P* = 0.025) was linked to poor prognosis, which could be an independent prognostic factor for the prognosis of OC patients. Moreover, top 100 genes screened by Pearson’s value associated with Hsp90, indicating that Hsp90 participated in the regulation of ATF5 target genes, PRAGC1A target genes and BANP target genes and also enriched in the metabolic processes of cell response to DNA damage stimulus, response to heat and protein folding.

**Conclusion:**

Hsp90 level is positively associated with OC mortality and is a potential prognostic indicator of OC.

## Introduction

OC is one of the most common gynaecological tumours. With approximately 239,000 cases being diagnosed and more than 150,000 deaths each year, OC is the main cause of death among women globally. According to the up-to-date report of the United States, 22,240 individuals were diagnosed with OC every year, and 14,070 died with OC [1]. Notably, the highest morbidity and mortality rates are also reported in Eastern and Central Europe [2]. Furthermore, the 5-year survival rate of OC patients in advanced remains at approximately 30% [3]. Approximately 60% of patients were diagnosed at advanced stages on account of the ovary is located deep in the pelvis, and the lack of novel biomarkers and therapeutic targets, and the ignorance of early screening in a large portion of patients [4]. Therefore, it has great significance to exploring the molecular mechanism of OC for the clinical treatment [5].

According to the OC Research Alliance (OCRA), the recurrence rates of FIGO stage I and II are 10% and 30% worldwide, while the recurrence rates of stage III and IV OC raised to 70%—95% [6]. Epithelial OC (EOC) is difficult to diagnose at the early stage, due to the depth of the pelvis epithelium. Therefore, as a result, approximately 60% of patients are diagnosed of terminal OC [4]. Despite receiving the neoadjuvant chemotherapy and aggressive radical surgery, the 5-year survival rate of advanced EOC patients is even less than 30% [7]. With the usual metastasis of local infiltration and the high postoperative recurrence rate, EOC patients need regular examination (to evaluate the potential progression of disease, with the routine test of tumour biomarkers and imaging examinations). However, the effectiveness of the examination and assessment of long-term survival outcome with the current tools are still inadequate. In addition to active clinical treatment, the screening, diagnosis, prognostic assessment, and recurrence monitoring of OC are important roles of clinical treatment.

As an ATP-dependent molecule, Hsp90 plays an important role in the maturation, stabilization and activation of different targeted proteins in cells. It is simultaneously involved in the folding, maturation and degradation of client proteins [8]. As a molecular chaperone, Hsp90 regulates the activation of various client proteins. This implicated that Hsp90 activity needs to be controlled by various coordinated regulatory mechanisms in participating in different biological processes [9]. Hsp90 plays an important regulatory role in the maturation of certain key signaling proteins, such as kinases [10–12], steroid hormone receptors [13] and transcription factors [14]. In addition, Hsp90 is also indispensable in the assembly and disassembly of protein complexes [15] and the suppression of phenotypic variation [16–19]. Previous studies have shown that heat shock protein (HSP) is overexpressed in a large amount of cancers, including cervical cancer, bladder cancer, breast cancer, OC, prostate cancer and so on [20]. Different subtypes of Hsp90 all lead to the poor prognosis of tumours. In addition, there studies suggested that Hsp90 can be used as a prognostic indicator in triple-negative and HER2/ER2 + tumours, which can increase the risk of recurrence and distant metastasis [21]. Moreover, extracellular Hsp90 (eHsp90) is associated with the transformation of epithelial cells to mesenchymal cells, which promotes the progression of cancer [22]. Epithelial–mesenchymal transition (EMT) is the typical morphological change in tumorigenesis, including the elongation of fibroblasts, the formation of loosely packaged phenotypes and the regulation of EMT effectors, as well as the function of EMT mediators, such as N-cadherin, Snail, Zeb1, Zeb2, Slug and MMP-9 [23]. Similar to Hsp27 and Hsp70, Hsp90 is an important protein in apoptosis by regulating p53, NF-κB, Akt, RAF-1, JNK and other apoptosis factors. Apoptosis can be blocked by further inhibiting the activation of caspase by combining with apAF-1 [24]. Moreover, inhibition of Hsp90 in mitochondria increases the permeability of transition pores, leading to the loss of membrane potential, blocking the transmission of the calcium-mediated stress response, and consequently inhibiting the process of cell apoptosis. This process leads to depletion of calcium in mitochondria and activation of another pro-apoptotic transcription factor, CHOP [25].

In our study, we measured Hsp90 concentrations in peripheral blood of 106 OC patients. Hsp90 level was measured to assess its potential association with the clinical and pathological features of patients. Finally, the effect of Hsp90 level on the prognosis of OC was tested according to disease-specific survival time. We found that the Hsp90 level is related to the disease-specific survival time of OC. In addition, the main targeted biological pathways and metabolic processes regulated by Hsp90 of OC patients were analysed by using bioinformatics analysis and GSEA and GSVA analysis for functional enrichment. Immunohistochemical methods were conducted to evaluate and compare the expression of Hsp90 in OC tissues of each FIGO staging.

## Materials and methods

### Clinicopathologic feature of patients and inclusion criteria

We conducted a retrospective study of the clinical data of 106 OC patients, who received inpatient clinical treatment at the First Affiliated Hospital of Anhui Medical University from January 2017 to August 2022. Information including age, occupation, contact phone number, discharge and admission dates, past medical history, image examination result, laboratory examination result, pathological data, and hospitalization number were collected. We collected peripheral blood at the first diagnosis of patients without any clinical treatment. By summarizing the clinical manifestations, we firstly screened age, Hsp90 level, survival time and outcome during the follow-up, FIGO stage, age of first birth, CA125 level, Case type, Ascites and Residual tumor for subsequent analysis. HE4 (human epididymal protein 4), a commonly used biochemical biomarker of OC, was also included in our study. The inclusion criteria: (1) patients with OC confirmed by postoperative pathology; (2) patients received radical surgery of OC. The exclusion criteria: (1) lack of clinical data; (2) the cause of death was not ovarian cancer; (3) complicated with other malignant tumours; (4) expected survival time less than 5 years or presence of other life-threatening serious diseases; (5) lack of follow-up records. Regarding the follow-up time and content, some patients were followed up in the outpatient clinic after surgery, and most of them were followed up by telephone for 2 to 5 years postoperatively. Patients were followed up for each 3 to 6 months, including the information of routine blood tests, biochemical tests, chest X-ray, abdominal and pelvic ultrasound or CT. The starting point of survival time was the date of diagnosis in our hospital, and the end point was the date of death or August 25, 2022, which with the median follow-up time is 28 months.

### Statistical analysis

SPSS (version 26.0) (SPSS, Chicago, IL, USA) was used for database establishment and statistical analysis, and *P* < 0.05 indicated the statistic significant. A total of 106 eligible OC patients were enrolled in the current study. During the 5-year follow-up period, 32 patients died of OC. Based on the results of 557 healthy men and women aged 18-78 years, the normal reference value of Hsp90 was 0-82.06 ng/mL (Data from the manufacturer: Protgen Company. Based on a clinical randomized study provided by the reagent manufacturer, the normal reference range of Hsp90 was established in the range of 0-82.06 ng/ml). According to the normal values provided by the reagent manufacturer, 54 patients were assigned to the normal Hsp90 level group, and 52 patients were assigned to the abnormal Hsp90 level group. The continuous variable of Hsp90 was converted into low or high level according to the data from the manufacturer. While the separation of HE4 was converted according to the data from the manufacturer (Roche Company, premenopause:0-70 ng/mL,postmenopausal:0-140 ng/mL). Moroever, the separation of CA125 was converted according to the data from the manufacturer (Siemens Healthcare Diagnostics Company, 0-30.2 ng/mL). According to previous studies, every 5-year delay in the age of first birth was associated with a 13% reduction in the risk of OC (95%CI, 5%-21%, *P* = 0.003). Those who gave birth for the first time after age 35 had a 47% lower risk of OC than those who gave birth before 25y [26]. Therefore, patients were divided into two groups according to the age of first birth (The age of first birth ≤ 24y group and > 24y group). The relationship between the clinicopathological characteristics of OC patients and survival status was analysed by the Chi-square test or Fisher’s exact test. The log-rank test of Kaplan‒Meier survival analysis and univariate survival analysis based on Cox proportional hazards model were used to estimate the survival analysis. HR and 95% CI were calculated by using the Cox proportional hazards model. The independent prognostic effect of the risk score was calculated by using multivariate Cox survival analysis.

### Bioinformatics analyses

First, RNA-sequence V2 RSEM data, which included 19,063 genes expressed in OC tissues, were downloaded from https://www.cbioportal.org/ by using the “cgdsr” R package. The top 100 genes positively correlated with Hsp90 level (Pearson’s correlation ≥ 0.3, *P* < 0.0001) were filtered by calculating Pearson’s correlation coefficients. Then, bioinformatics analyses of functional signalling pathways were performed via Metascape, an online platform (https://metascape.org/). A q-value < 0.05 was used as the threshold for indicating significance.

### GSEA (gene set enrichment analysis) and GSVA (gene set variation analysis) analysis of Hsp90

The gene expression profile of TCGA-OV was obtained through UCSC Xena, which can be accessed via this link: https://xenabrowser.net/datapages/?dataset=TCGA.OV.sampleMap%2FHiSeqV2_PANCAN&host=https%3A%2F%2Ftcga.xenahubs.net&removeHub=https%3A%2F%2Fucscpublic.xenahubs.net. In order to investigate the mechanism of tumorigenesis, we utilized gene set enrichment analysis (GSEA) to explore the activated signaling pathways in patients with both high and low levels of HSP90AA1. To obtain the necessary 186 Kyoto Encyclopedia of Genes and Genomes (KEGG) pathway information, we downloaded the relevant data from MSigDB (https://www.gsea-msigdb.org/gsea/msigdb/genesets.jsp?collection=CP:KEGG) [27–29]. One way to describe biological features within KEGG gene sets is through enrichment analysis. This method calculates a normalized enrichment score (NES) that indicates the level of pathway activation. To further evaluate pathway activities among patients with varying levels of HSP90AA1, we utilized gene set variation analysis (GSVA) with the "GSVA" R package. Additionally, we obtained 50 hallmark gene sets from MSigDB for this analysis.

### Enzyme-linked immunosorbent assay

Serum was extracted from the patient’s peripheral circulating blood and stored at 2–8 °C. The blood was timely tested on the day the blood was taken. We used a commercial PROTGEN ELISA kit (for the detection of Hsp90).

The kit was equilibrated at 37 °C for 30 min. The liquid was thoroughly mixed to avoid foaming before use. Then, 475 ml of deionized water was added to the concentrated washing solution and mixed well. The calibrator was added to 0.4 ml analyte diluent to dissolve evenly, and the test sample was diluted 20 times with diluent. Then, the required number of strips was placed on the plate rack, the calibration well and sample well were set, and 50 µl of calibrated and diluted samples were added. Then, 50 µl of the Hsp90 marker solution was added to each microwell and gently shaken evenly. Next, the microporous plate was covered with a sealing plate and incubated at 37 °C for 60 min. After incubation, the reaction solution was removed, 300 µl of detergent was added to each well to wash the plate for a total of 6 washes, and the plate was finally dried on absorbent paper. Then, 50 µl of chromogenic agent A and liquid B were added to each well once, shaken gently and evenly, and incubated at 37 °C for 20 min. Then, 50 µl of stop solution was added to each well to stop the colour development. Within 10 min after adding the reaction stopping solution, the DR-200B series enzyme tag analyser was used to read the OD value at 450/620 (630) nm wavelength. Instrument software was used to draw the standard curve with the logarithm of the concentration of calibration products 1–5 as the X-axis and the logarithm of the light absorption value of the calibration product as the Y-axis. The logarithm of sample absorbance was inserted into the regression equation to calculate the content of Hsp90 in the sample. (The logarithmic fitting curve is recommended, and the correlation coefficient R^2^ of the standard curve should be greater than 0.980) [30].

### Immunohistochemistry (IHC) staining for Hsp90

To evaluate the expression of Hsp90 in ovarian cancer tissues, we randomly selected 20 patients from the included patients with FIGO stages of I, II, III, and IV of 5. IHC staining was used to validate the immunophenotype-types in the FIGO cohort. Hsp90 polyclonal antibody (Cat:No.13171–1-AP, Proteintech Group, Inc Suite 400, Rosemont, USA) was employed to reflect stromal activation and distinguish the immune-activated and immune-suppressed sub-types. The detailed steps of the IHC procedure havebeen previously reported [31]. Hsp90 is commonly expressed in the cytoplasm of ovarian cancer cells. We used a positive staining area score (0, negative; 1–10%; 2, 11–50%; 3, 51–80%; 4, > 80% positive area) multiplied by the immunostaining intensity score (0, unstained; 1, weak; 2, mild; 3. Strength) semi-quantified the results. The whole evaluation process was conducted by two experienced pathologists who were unaware of the information of the patients. We averaged the evaluated results as the final result.

## Result

### Correlation between the clinical characteristics and the prognosis of enrolled patients

The correlation between OC prognosis and clinicopathologic feature was analyzed by chi-square analysis. The results revealed that the poor prognosis of patients was significantly correlated with the abnormal Hsp90 level and the high FIGO stage of tumors (Hsp90: χ^2^ = 13.552, *P* = 0.000; FIGO stage: χ^2^ = 6.3847, *P* = 0.009; Case type: χ^2^ = 9.307, *P* = 0.005; Ascites: χ^2^ = 6.805, *P* = 0.017 Table 1). HE4, as a biochemical index of OC commonly used in clinical work, has a certain correlation with the prognosis of OC patients (χ^2^ = 3.643, *P* = 0.056 Table 1).

**Table 1:** The prognosis and characteristics of the included patients

Characteristics	total	Death	Survival	p	χ^2^
Summary	106	32	74		
Age					
≥ 60	52	13	39	0.253	1.304
< 60	54	19	35		
Hsp90 expression					
Abnormal	54	25	29	0.001*	13.552
Normal	52	7	45		
Stage of cancer (FIGO)					
IV and IIIC recurrent	78	29	49	0.009*	6.847
IIIC and less than IIIC	28	3	25		
Age of first birth					
≥ 24y	50	14	36	0.556	0.347
< 24y	54	18	36		
Childless	2	0	2		
HE4					
Abnormal	58	22	36	0.056	3.643
Normal	48	10	38		
CA125					
Abnormal	89	27	62	0.939	0.006
Normal	17	5	12		
Case type					
Serous carcinoma	83	31	52	0.005*	9.307
Other	23	1	22		
Ascites					
Yes	74	28	46	0.017*	6.805
No	32	4	28		
Residual tumor					
Yes	56	18	38	0.801	0.215
No	50	14	36		

^*^P < 0.05

### Abnormal Hsp90 level was indicated poor prognosis

**Fig. 1:**
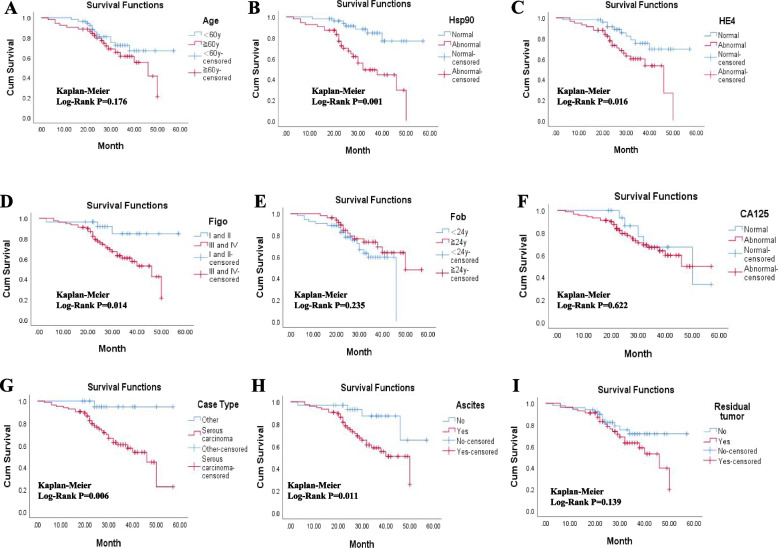
Differential clinical outcome of different subgroups. **A** Kaplan–Meier survival analysis of the Age group; **B** Kaplan–Meier survival analysis of the Hsp90 expressin group; **C** Kaplan–Meier survival analysis of the HE4 expression group; **D** Kaplan–Meier survival analysis of the FIGO stage group; **E** Kaplan–Meier survival analysis of the Age of first birth group; **F** Kaplan–Meier survival analysis of the CA125 group; **G** Kaplan–Meier survival analysis of the Case type group; **H** Kaplan–Meier survival analysis of the Ascites group; **I** Kaplan–Meier survival analysis of the Residual tumor group

To further explore the prognostic value of these features, Cox univariate analysis (Table 3) and Kaplan‒Meier univariate analysis (Fig. 1) were performed to evaluate the prognostic value of clinical characteristics of the enrolled patients. We found that patients with different age and age of first birth did not show different disease-specific survival time (Fig. 1A, E). Patients in the abnormal Hsp90 level group had a significantly shorter average survival time as compared to those in the normal Hsp90 level group (50.781vs. 34.559 months, Log-Rank *P* < 0.001, Fig. 1B, Table 2). Meanwhile, HE4 group, FIGO group, Case type group and Ascites group also showed similar differential changes (HE4: 48.188 vs. 36.592 months, Log-Rank *P* = 0.016, Fig. 1C, Table 2; FIGO stage: 38.010 vs. 51.649 months, Log-Rank *P* = 0.014, Fig. 1D, Table 2; Case type: 39.543 vs. 55.263 months, Log-Rank *P* = 0.006, Fig. 1G, Table 2; Ascites: 37.837 vs. 50.067 months, Log-Rank *P* = 0.011, Fig. 1H, Table 2).

**Table 2:** The mean survival time of different subgroups

Parameters	Mean Survival Time
Age	
< 60y	46.791
≥ 60y	37.876
Hsp90	
Normal expression	50.781
Abnormal expression	34.559
HE4	
Normal expression	48.188
Abnormal expression	36.592
FIGO	
Stage I and II	51.649
Stage III and IV	38.010
Age of first birth	
< 24y	36.402
≥ 24y	45.157
CA125	
Abnormal	42.908
Normal	45.113
Case type	
Serous carcinoma	39.543
Other	55.263
Ascites	
Yes	37.837
No	50.067
Residual tumor	
Yes	38.199
No	47.251

Moreover, univariate survival analysis of Cox proportional hazards model was conducted for further analysis of prognostic value of Hsp90 level and other clinical parameters. The result suggested that Hsp90 was significantly correlated with the poor prognosis of OC patients (HR = 4.618,95% CI = 1.988–10.727, *P* < 0.001, Table 3).

**Table 3:** Cox univariate survival analysis of different subgroups

Variables	B	SE	Wald	df	Sig	Exp(B)	95.0% CI for Exp(B)	
							**Lower**	**Upper**
Age	0.480	0.360	1.777	1	0.182	1.616	0.798	3.275
Hsp90	1.530	0.430	12.659	1	0.001*	4.618	1.988	10.727
FIGO	-1.383	0.608	5.166	1	0.023*	0.251	0.076	0.827
HE4	-.898	0.386	5.396	1	0.020*	0.408	0.191	0.869
Age of first birth	0.433	0.369	1.376	1	0.241	1.542	0.748	3.180
CA125	0.241	0.492	0.240	1	0.624	1.273	0.485	3.340
Case type	2.284	1.018	5.031	1	0.025*	9.816	1.334	72.220
Ascites	1.276	0.526	5.664	1	0.017*	3.582	1.253	0.247
Residual tumor	0.542	0.373	2.114	1	0.146	1.719	0.828	3.567

^*^P < 0.05

### Hsp90 level: a risk factor for OC patient prognosis independently

Multivariate Cox survival analysis was performed to eliminate the false positive results. In order to exclude the influence of other clinical features on the outcome, we screened out the positive clinical features of the Cox univariate survival analysis for Cox multifactorial survival analysis. Cox multivariate analysis results revealed that abnormal Hsp90 level (HR = 2.710, 95% CI = 1.087–6.757, *P* = 0.032, Table 4) could be regarded as an independent risk factor for the poor prognosis of OC patient.

**Table 4:** Cox multivariate survival analysis of clinical parameters

Variables	B	SE	Wald	df	Sig	Exp(B)	95.0% CI for Exp(B)	
							**Lower**	**Upper**
Hsp90	0.997	0.466	4.575	1	0.032	2.710	1.087	6.757
HE4	0.314	0.415	0.570	1	0.450	1.369	0.607	3.092
FIGO	0.279	0.800	0.122	1	0.727	1.322	0.276	6.344
Case type	1.366	1.075	1.615	1	0.204	3.920	0.476	40.621
Ascites	0.392	0.696	0.317	1	0.573	1.480	0.379	5.791

### Different distribution of clinicopathological characteristics among abnormal and normal Hsp90 level subgroups

We further analysed the clinical parameters of OC patients and investigated their correlation with Hsp90 level (Table 5). There was no significant difference between two subgroups of the dignosis age (*P* = 0.843, Table 5), the age of first birth (*P* = 0.838, Table 5), CA125 (*P* = 0.539, Table 5) and Residual tumor (*P* = 0.849, Table 5) in general clinical characteristics. However, by chi-square analysis, we found that the level of Hsp90 was correlated with the level of HE4, FIGO stage, survival outcome, Case type and Residual tumor of patients (HE4, *P* = 0.000 χ^2^ = 16.646; FIGO stage, *P* = 0.001 χ^2^ = 10.248; Survival *P* = 0.000 χ^2^ = 20.161 Table 5; Case type, *P* = 0.001 χ^2^ = 16.883; Ascites, *P* = 0.001 χ^2^ = 19.010).

**Table 5:** Correlation between Hsp90 expression level and clinical parameters

Characteristics	total	Abnormal expression	Normal expression	*p*	χ2
Summary	106	54	52		
Age					
≥ 60	52	27	25	0.843	0.039
< 60	54	27	27		
Overall survival					
Death	32	25	7	0.001*	20.161
Survival	74	19	45		
Stage of cancer (FIGO)					
IV and IIIC recurrent	78	47	31	0.001*	10.248
IIIC and less than IIIC	28	7	21		
Age of first birth					
≥ 24y	50	26	24	0.838	0.042
< 24y	54	27	27		
Childless	2	1	1		
HE4					
Abnormal	58	40	18	0.001*	16.646
Normal	48	14	34		
CA125					
Abnormal	89	47	42	0.539	0.773
Normal	17	7	10		
Case type					
Serous carcinoma	83	51	32	0.001*	16.883
Other	23	3	20		
Ascites					
Yes	74	48	26	0.001*	19.010
No	32	6	26		
Residual tumor					
Yes	56	31	25	0.849	0.036
No	50	23	27		

^*^P < 0.05

### Bioinformatics analyses showed the biological processes involved and major target gene pathways affected

**Fig. 2:**
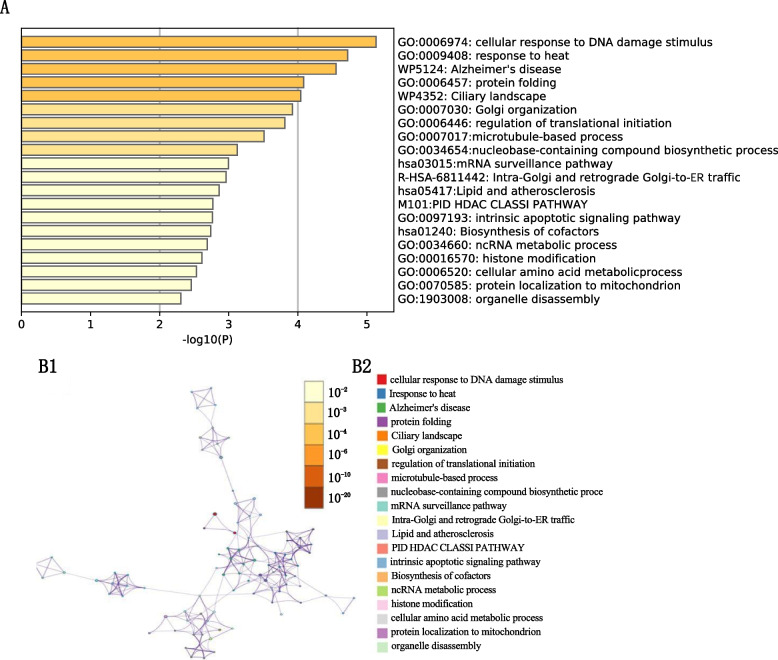
Pathway enrichment showing Hsp90 might impact biological pathways

**Fig. 3:**
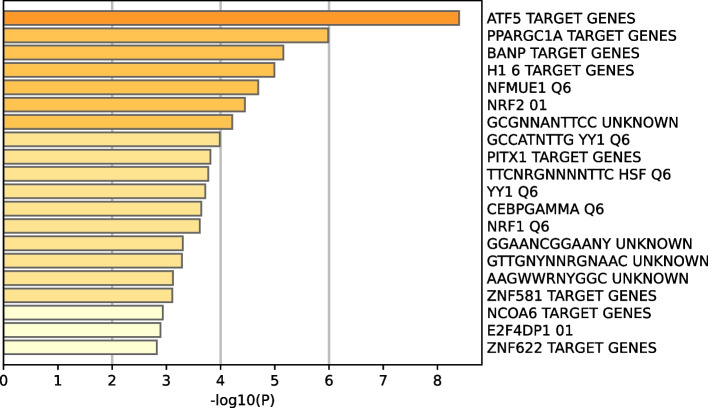
Transcript factors that might regulate Hsp90 expression

We performed functional enrichment analysis to explore the biological pathways and processes correlated with HSP90AA1, the main coding gene of Hsp90. First, by calculating Pearson’s correlation coefficients between HSP90AA1 expression and other mRNA based on the TCGA dataset. In the aggregate that 100 genes with the highest correlation value were selected (Pearson’s correlation ≥ 0.3, *P* < 0.0001). Pathway and process enrichment analyses were carried out with the following ontology sources for each gene list: KEGG Pathway, GO Biological Processes, Reactome Gene Sets, Canonical Pathways, Cell Type Signatures, CORUM, TRRUST, DisGeNET, PaGenBase, Transcription Factor Targets, and WikiPathways [32]. The pathways impacted by HSP90AA1 were mainly enriched in the activation of extracellular related biological processes, including cell response to DNA damage stimulus, response to heat, Alzheimer’s disease, and protein folding (Fig. 2). To further analyze the related gene target pathways regulated by HSP90AA1, we conducted enrichment analysis on the targets of transcription factors, and the results indicated that the target pathways of the included genes were mainly enriched in ATF5 target genes, PRAGC1A target genes and BANP target genes (Fig. 3).

### Hsp90 interacted biological pathways

**Fig. 4:**
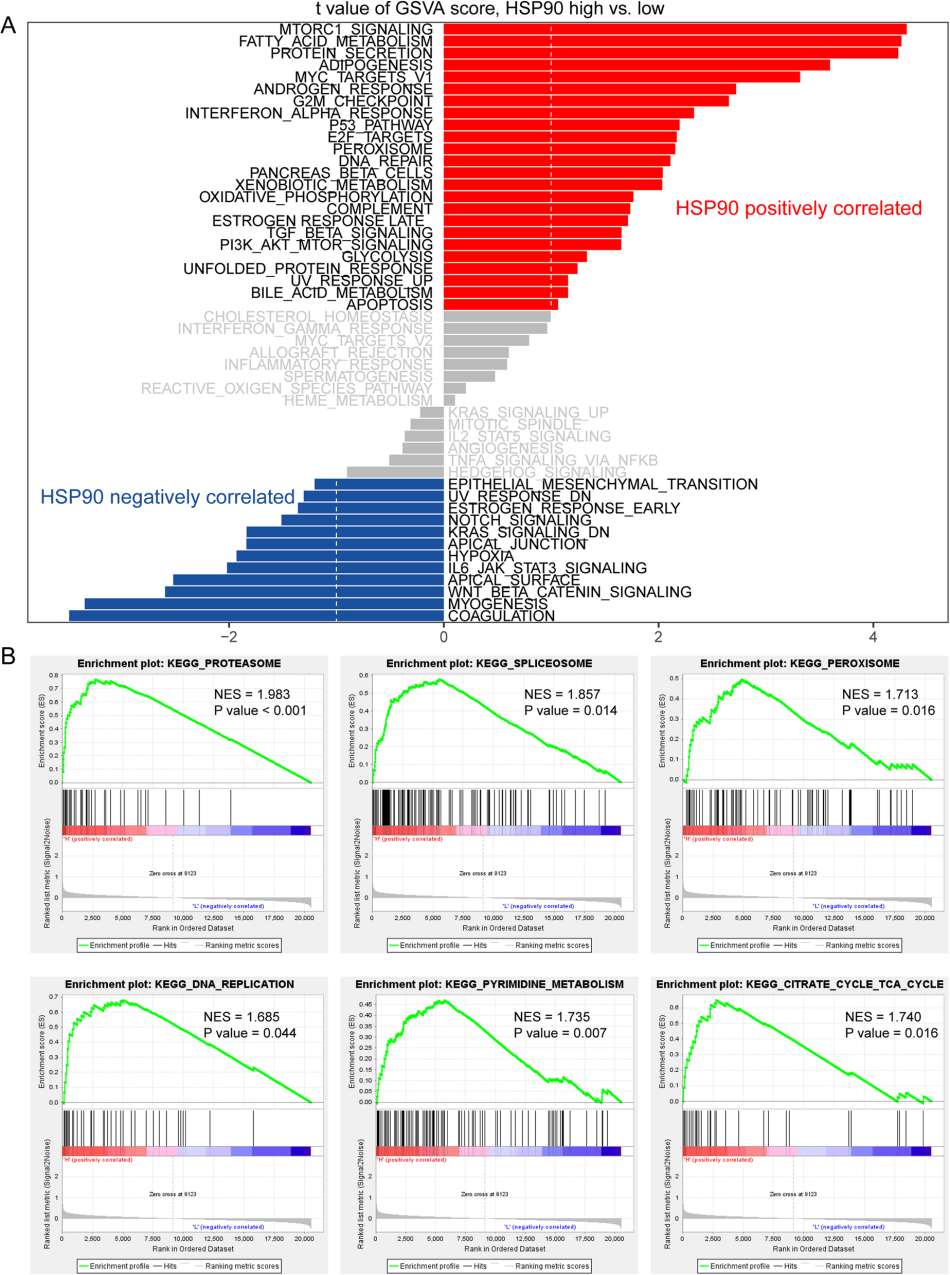
**A** Hsp90 positively correlated pathways by GSVA; **B **Hsp90 positively correlated pathways by GSEA

To investigate how Hsp90 impacts OV at a deep mechanistic level, we utilized the GSEA and GSVA algorithms to identify the most significantly altered pathways. Specifically, for GSVA, we compared 50 hallmark gene sets to identify activated tumor-associated pathways, and our findings suggest a positive correlation between HSP90AA1 expression and the activation of several biological processes involved in cell cycle regulation, including MTORC1 signaling, MYC targets, G2M checkpoint, and E2F targets. Moreover, the activation of pathways involved in fatty acid metabolism, protein secretion, peroxisome function, and oxidative phosphorylation was also observed, reflecting active cell proliferation events (Fig. 4A). Our GSEA analysis of 186 KEGG terms yielded similar results, including the activation of pathways involved in proteasome function, spliceosome activity, peroxisome function, DNA repair, pyrimidine metabolism, and citrate cycle TCA cycle (Fig. 4B).

### Hsp90 is widely expressed in ovarian cancer cells

**Fig. 5:**
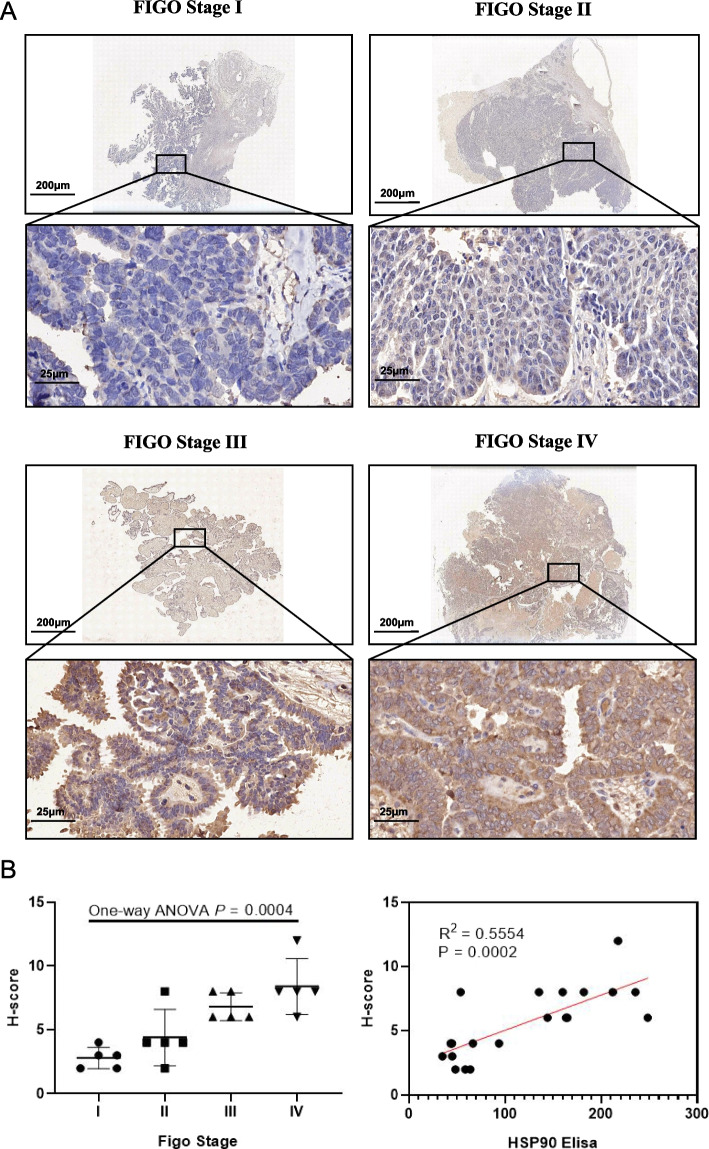
**A** Expression of Hsp90 in ovarian cancer tissue with different FIGO stages; **B** Immunohistochemical H-score was significantly correlated with FIGO stage and peripheral blood Hsp90 level

Immunohistochemical methods were used to evaluate and compare the expression of Hsp90 in 20 OC tissues (we randomly selected 5 cases of each FIGO stage). We found that Hsp90 is widely expressed in the cytoplasm of ovarian cancer cells (Fig. 5A). The H-score of Hsp90 showed that it was significantly correlated with FIGO stage (*P* = 0.0004) and peripheral blood Hsp90 concentration (R^2^ = 0.5554, *P* = 0.0002) (Fig. 5B).

## Discussion

OC as the most frequent tumour types of gynecological malignancies worldwide, improving the early diagnosis rate of OC has aroused wide public concern. Seventy to ninety percent of patients with OC were recurrent after initial treatment in 2 years. As a result, identifying novel biomarkers and therapeutic targets to improve the prognosis of OC patients is of great importance. Regarded as a highly evolutionarily conserved chaperone, Hsp90 is a deep-studied member of the heat shock protein family. Hsp90 is highly expressed in tumor tissues, and promoted cell growth, proliferation and survival by participate in the continuous activation of various cellular kinases and transcription factors in malignant cell stress [33].

In our study, the level of Hsp90 in OC patients was analysed based on peripheral blood samples from selected patients at the first diagnosis of patients without any clinical treatment. The clinical characteristics of OC patients were collected, including age, survival period after diagnosis, tumour FIGO stage, age of first birth, CA125 level, Case type, Ascites, Residual tumor and HE4, which are currently widely used as clinical OC indicators. HE4 is showed low levels in the epithelium of respiratory and reproductive tissues, including OC, compared to high levels in OC cells [34]. High secretory levels were also found in the serum of OC patients [35]. Related studies suggested that HE4 could be an important early indicator of OC recurrence [36]. First, we carried out chi-square analysis on the survival and clinical manifestations of the patients during the follow-up period, and the results suggested that the prognosis of the patients was related to the abnormal Hsp90 level, the abnormal HE4 level, the FIGO stage, Case type and Ascites. We further performed Kaplan‒Meier univariate survival analysis and univariate survival analysis of Cox’s proportional hazards model for the clinical features of the study. We found that Hsp90 level, HE4 level, higher FIGO stage, Case type and Ascites may lead to poor prognosis with shorter mean survival time in OC patients. A chi-square test for the relationship between Hsp90 level levels and other clinicopathological features was further performed, the result suggested that abnormal Hsp90 level was related with abnormal HE4 level, higher FIGO stage, survival, Case type and Ascites. By conducting multivariate analysis of the Cox proportional hazards regression model, we eliminated false positive results produced by the interaction of different clinicopathological features. The results revealed that the Hsp90 level could be an independent prognostic factor for the prognosis of OC patients. Immunohistochemical methods were conducted to evaluate and compare the expression of Hsp90 in OC tissues of each FIGO stage. The result showed that Hsp90 level in ovarian cancer tissue was significantly correlated with FIGO stage and peripheral blood Hsp90 concentration. Therefore, we hypothesized that Hsp90 could be an oncogene of OC, and the treatment of OC by Hsp90 inhibitor is feasible.

In previous studies, the high level of Hsp90 in a large amount of cancer tissues has been widely revealed [37–40]. According to research in the last 10 years, 82.7% of lung cancer samples showed moderate and high Hsp90 level [41], 50% high Hsp90 level of the Undifferentiated pleomorphic sarcoma [42], Hsp90 was expressed in in 63.5% samples of the breast cancer [43], 50% high Hsp90 level of the gastric cancer [44], over expression of Hsp90 was observed in 76.74% of non muscle invasive bladder cancer samples [45], but studies on Hsp90 level in OC have not been defined. As a secretable protein [46], the level of Hsp90 in serum of OC patients can also be used in liquid biopsies. Because of its importance in regulating different cellular proteins, researchers have attempted to use Hsp90 inhibitors to treat different cancers [47]. Since the first Hsp90 inhibitors in clinical research in the 1990s, numerous pharmaceutical companies have invested great enthusiasm and energy, and researchers have adopted combinations, alone or with indications of a variety of different development strategies, such as switches, with more than 30 years and nearly 30 clinical trials of candidate drugs. More than 20 candidates have announced clinical failures. Many scientists have been working on the application of Hsp90 inhibitors in cancer treatment, from first-generation drugs (Geldanamycin (GA) and Radicicol (RD)) [48] to second-generation drugs (related derivatives of GA and RD) [49]. But the results of clinical trials were unsatisfactory, and unfortunately none of these drugs were approved as new drugs [50]. Therefore, the role of Hsp90 in the occurrence and development of cancer is still a hot topic of research. Consequently, the effect of Hsp90 inhibitors in clinical trials has not been satisfactory thus far, and the mechanism of Hsp90 needs further study.

Therefore, in this study, 100 genes positively correlated with Hsp90 level in OC were screened. Through bioinformatics methods, we used the following ontology sources: KEGG Pathway, GO Biological Processes, Reactome Gene Sets, Canonical Pathways, Cell Type Signatures, CORUM, TRRUST, DisGeNET, PaGenBase, Transcription Factor Targets. Functional enrichment analysis was performed to explore biological pathways and processes related to Hsp90. According to the results, these 100 genes are concentrated in the following biological processes: cell response to DNA damage stimulus, response to heat, Alzheimer’s disease, and protein folding. Furthermore, enrichment analysis of the target targets of transcription factors indicated that the target target pathways of the included genes were mainly enriched in ATF5 target genes, PRAGC1A target genes and BANP target genes. Consistent with our results, previous investigation about bortezomib showed that in the process of inducing apoptosis, ATF5 and Hsp90 have synergistic effect [51]. To investigate how Hsp90 impacts OV at a deep mechanistic level, we utilized the GSEA and GSVA algorithms to identify the most significantly altered pathways.Our GSVA result suggested a positive correlation between Hsp90 level and the activation of several biological processes involved in cell cycle regulation, including MTORC1 signaling, MYC targets, G2M checkpoint, and E2F target. Moreover, the activation of pathways involved in fatty acid metabolism, protein secretion, peroxisome function, and oxidative phosphorylation was also observed, reflecting active cell proliferation events.Our GSEA analysis of 186 KEGG terms yielded similar results, including the activation of pathways involved in proteasome function, spliceosome activity, peroxisome function, DNA repair, pyrimidine metabolism, and citrate cycle TCA cycle. Hsp90 has also been reported as a new target protein of CDDO-Me [52]. It has been suggested that Hsp90 in mitochondria blocks the transmission of the calcium mediated stress response, which inhibits the apoptosis of cancer cells. Inhibition of mitochondrial Hsp90 increases the permeability of transition pores and membrane potential loss. This then releases calcium from the mitochondria, whose consumption eventually leads to the activation of another pro-apoptotic transcription factor, CHOP [25].

In our study, we constructively proposed the use of peripheral blood samples as a low-cost detection method in order to benefit more patients. Our results also confirmed that the expression level of HSP90 is correlated with the prognosis of OC patients, but our study still has the following limitations.Firstly, although we selected all ovarian cancer patients in our hospital in the past 5 years as research objects, but after screening, we only got 106 patients who met the inclusion criteria. Secondly, Our follow-up time was also relatively inadequate (median follow-up time was 28 months). Thirdly, our study is a single-center study, and regional differences may cause certain bias in our results.

In addition, we also performed immunohistochemical validation in real-world sample to confirm the expression of HSP90 in OC tissue, and further investigated the potential mechanisms of HSP90 by bioinformatics. Immunohistochemical results indicated that HSP90 was widely expressed in ovarian cancer tissues, and the expression concentration increased with the increase of FIGO stage. The expression level of HSP90 was positively correlated with that of HSP90 in peripheral blood. Bioinformatics attempts to elucidate the possible mechanism of HSP90 in the development of ovarian cancer. The functional enrichment analysis illustrates the signaling pathways and biological processes affected by HSP90. The GSEA and GSVA algorithms to identify the most significantly altered pathways. We further revealed the biological function of Hsp90 and the regulatory mechanism in the internal environment, which provides a basis for the clinical drug use of OC.The above studies are consistent with the results of our peripheral blood studies. However, it is expected that further optimization and improvement will be carried out in the follow-up research.

## Conclusions

In general, we found that the Hsp90 level in the blood of OC patients was associated with lower OC patient disease-specific survival time. The level of Hsp90 is an independent risk factor for the prognosis of OC patients, even after adjusting of HE4 value,FIGO tumor stage, Case type and Ascites. These results suggested that Hsp90 can improve the prognostic assessment of OC in the clinic treatment.

## Data Availability

All data generated and analyzed during this study are included in this manuscript. The datasets generated and analyses during the current study are available from the corresponding author on reasonable request.

## Abbreviations

OC: Ovarian cancer
Hsp90: Heat shock protein 90
HE4: Human epididymal protein 4
OCRA: Ovarian cancer Research Alliance
EOC: Epithelial ovarian cancer
EMT: Epithelial–mesenchymal transition
FIGO: The International Federation of Gynecology and Obstetrics
GSEA: Gene set enrichment analysis
GSVA: Gene set variation analysis
KEGG: Kyoto Encyclopedia of Genes and Genomes
GA: Geldanamycin
RD: Radicicol
